# The effect of relationship marketing towards switching barrier, customer satisfaction, and customer trust on bank customers

**DOI:** 10.1186/s13731-023-00270-7

**Published:** 2023-05-05

**Authors:** Kadarisman Hidayat, Muhammad Ishlah Idrus

**Affiliations:** 1 Faculty of Administrative Science, University of Brawijaya, Malang, Indonesia; 2 Department of Accounting, Faculty of Economics, State Polytechnic of Ujung Pandang, Makassar, Indonesia

**Keywords:** Relationship marketing, Switching barrier, Customer satisfaction, Customer retention, SEM (structural equation modelling)

## Abstract

Basically, relationship marketing focuses more on creating customer value through interaction with customers to get information regarding evaluation of customer needs and expectations on an ongoing basis. Interaction with customers must be conducted, because customer involvement can increase customer value, so that the company can meet their expectations and needs. Furthermore, customer satisfaction, customer trust, and customer retention can be influenced by the implementation of a relationship marketing strategy. This study aims to examine and analyze the correlation between relationship marketing variables to switching barriers, customer satisfaction, customer trust and customer retention. Regarding to the objectives and study hypotheses, structural equation technique (SEM) is considered as relevant to be used. The population in this study was BNI customers who are members of BNI Emerald in East Java Province. The sample was obtained based on the top five BNI branches. Furthermore, the sample was determined by area proportional random sampling based on branches with a total sample of 141 respondents. Based on the study results, it is concluded that the effect of Relationship Marketing on Switching Barriers, Customer Satisfaction, and Customer Trust is positively significant. As a result, relational marketing is positioned as the primary exogenous variable to be investigated in conjunction with other relevant factors, such as switching barrier variables, customer happiness, customer trust, and customer retention. Customer satisfaction has a considerable positive impact on customer trust, indicating that the better the customer satisfaction, the higher the customer trust. Customer satisfaction has a positive and considerable impact on customer retention, indicating that the better the customer satisfaction, the higher the customer retention.

## Introduction

Relationship marketing focuses on creating customer value through engagement with customers, so that information about consumer requirements and expectations may be gathered on a continuous basis. According to Varey (2002), customer engagement is vital, since their participation may boost customer value, allowing the organization to satisfy their expectations and demands. Furthermore, the execution of a relationship marketing strategy can affect customer happiness, customer trust, and customer retention.

To succeed in today's hypercompetitive marketplaces, customers must be placed at the core of every company's operation. As a result of this customer-centered strategy, companies who wish to keep their current customers and attract new ones should acknowledge the vital and central role of human and subjective factors in the connections they've built with them. That is, value creation for and with customers must be directed first and foremost by the development of a strong cognitive and emotional relationship between consumers and businesses, with the ultimate objective of getting people "involved" in the activities of businesses (Rossi & Magni, 2017). Customer satisfaction, according to Ranaweera and Prabhu (2003), is one of the most important variables that might contribute to customer retention, trust, and switching obstacles. Furthermore, client retention is influenced by trust and switching obstacles. Lee et al. (2001) found similar results in another research.

According to another study by Lee et al. (2001), switching obstacles have a considerable impact on customer retention. To put it another way, if the switching barrier is strong, the firm will be able to keep its clients even if they are unhappy. In this situation, it may also be understood to mean that a firm must pay attention to elements such as switching barriers, trust, and relationship marketing to preserve its long-term existence.

BNI's aims for maintaining customer loyalty are to build strong relationships with customers and to achieve customer happiness. With fierce rivalry among Indonesian banks, BNI works to develop new marketing techniques on a regular basis to keep its consumers.

The growth in the Third Party Funds (DPK) from Rp. 230.08 trillion in September 2009 to Rp. 257.02 trillion in September 2010 was one of its achievements. Customer savings, which continued to rise, were one of the contributing elements to the increase in Third Party Funds. In addition, compared to 2010, the customer loyalty index was raised by one level in 2011. In comparison to other banks, this circumstance placed BNI in third place. Given the importance of customer savings in calculating the customer loyalty index, BNI is attempting to develop a marketing plan to maintain client loyalty.

Private client services (personal banking), personal advisory services (legal, education, and health consulting), financial planning (support customers' financial planning), personal investment services (giving advice on investments), personal debt and asset management (managing asset portfolios), and personal customer services are the six categories of services currently provided by BNI to its Emerald customers. All of these product characteristics are provided to strengthen client connections and deliver a sense of contentment. Good connections and customer pleasure, according to the model used in this study, are thought to be able to build consumer trust. In the banking world, trust is the most important factor in gaining client loyalty, which leads to customer retention.

## Literature review

### Marketing concept

Managerial studies have traditionally seen socioeconomic settings as "isolated" areas attempting to protect themselves from the "external environment." As a result, a number of managerial models have been created to assist employees and decision-makers in safeguarding "internal resources" as a method of ensuring companies' long-term viability. Using the interpretive lens provided by a resource-based view and relationship marketing to investigate the impact of employees' perceptions of internal organizational assets and environmental dynamics on employees' orientation to knowledge hiding as a way to protect individual knowledge, the paper builds on this broad and widely accepted assumption. It is vital to boost the value of information through the establishment of human-centered management models and research routes to assist employees in overcoming their perceptions of isolation based on socioeconomic circumstances (Caputo et al., 2021).

In the marketing literature, relationship marketing has recently gotten a lot of attention. 'Attracting, retaining, and—in multi-service organizations—strengthening client connections' was the definition of 'relationship marketing' when it was initially presented (Berry, 1983). It was later stated as a new-old notion (Berry, 1995), with the addition that "the idea of a firm gaining the favor and loyalty of consumers by meeting their desires and needs was not unknown to the earliest of merchants." Simply put, relationship marketing is concerned with preserving ties between marketing, quality, and customer service to gain and keep consumers (Christopher et al., 1991; Gronroos, 1994).

Marketing philosophy has evolved from an internal orientation (inward-looking) to an external orientation (outward-looking). Internal orientation is reflected in the concepts of production, product, and sales, while external orientation is reflected in the marketing and social marketing concepts. However, each concept has its own uniqueness and application context (Cravens & Piercy, 1994; Kotler, 2003).

Marketing concept considers that the key for bringing organizational goals into realization lies in its ability to create, deliver and communicate customer value to its target market more effectively than customers and what it provides. Marketing concept lies on four main pillars: target markets, customer needs, integrated marketing, and profitability. The target market is the customer selected to be served with a marketing program specially designed for them.

### Relationship marketing

Basically, relationship marketing is an alternative strategic towards the traditional marketing mix approach as a way for gaining a sustainable competitive advantage (SCA) and the best way to retain customers in the long term (Litle & Marandi, 2003). This is consistent with Gronroos’s definition (1994). Relational marketing is an attempt to closely know each customer, create two-way communication with consumers, and manage mutual relationships between customers and consumers. In Yau et al. (1999), there was suggested that the current marketing philosophy has shifted from transactional marketing to Relationship Marketing which is often referred to as Relationship Marketing Orientation. It highlights the significance of perceived value and duration of customer support in the relationships between these customer-focused efforts and trust (Roberts & Petzer, 2021).

Researchers determined that a billion pupils used digital channels during the COVID-19 epidemic, showing the importance of digital technology in education. This paper investigates the role of e-learning practices in a knowledge transfer environment, such as the university, taking into account that student satisfaction refers to a short-term attitude resulting from an evaluation of the educational experiences lived and that the perceived quality of an educational background is a result of student satisfaction. Specifically, the research provides some particular findings through an exploratory analysis, evaluating students' happiness in terms of student-to-student contact, technology, and unique material. The findings offer insight on e-learning outcomes and student happiness, demonstrating how digital technologies are revolutionizing the educational experience (Magni & Sestino, 2021).

Relationship marketing has had a significant influence on the marketing discipline, resulting in a paradigm shift away from transactional marketing and toward relationship marketing (Kotler, 1992; Webster, 1992). 'Transaction marketing of the 1980s focused on individual sales; relationship marketing of the 1990s focuses on individual customers and aims to develop a long-term relationship between client and firm,' according to one explanation (Payne, 1994). As a result of this transition, new marketing definitions are developing that focus on connections rather than marketing mix components. 'Marketing is to build, maintain, and strengthen connections with consumers and other partners at a profit, so that the parties involved's objectives are satisfied,' according to a standard definition (Gronroos, 1994).

This is accomplished through the sharing of information and the keeping of commitments.' Services marketing (Gummesson, 1999), the network approach to industrial marketing (Hakansson, 1982), and quality management (Edvardsson et al., 1994) all have their roots in relationship marketing. A comprehensive examination of the evolution of relationship marketing is beyond the scope of this article. 1 However, it's worth noting that four elements (Berry, 1995) have fueled contemporary interest in relationship marketing: the maturation of services marketing, the prospective advantages of relationship marketing, consumer benefits, and technology advancements. Because of the intangibility of the service product, organizations aiming to differentiate their offers have placed a premium on relationship difficulties. Indeed, some writers argue that relationship marketing is one of the most effective ways for service organizations to gain a long-term competitive advantage (Czepiel, 1990; Perrien & Ricard, 1995). Relationship marketing has grown interwoven with a rising emphasis in service quality (Gronroos, 1983; Langeard & Eiglier, 1987).

Jones et al. (2000), briefly define switching barrier as any factor made by a company, so that customers find difficulty to switch to other providers or service providers. Switching barriers are essential for the management of service delivery and product development of the competing businesses based on the service provision. This is due to the fact that customers in an increasingly global marketplace are constantly assessing their options regarding the best possible service delivered to them by service providers. Therefore, if companies wish to develop long-term relationships with their customers, it is important to understand why dissatisfied customers may not switch to another company and how the presence of switching barriers can affect the evaluation of a company’s service recovery efforts (Yanamandram & White, 2006).

There was unanimous agreement that the Internet had a key role to play in relationship management but there was far less agreement about the rates of customer adoption and the extent to which this could or should be influenced by bank strategies (Durkin & Howcroft, 2003). Many banks have already reached a higher level of relationship marketing than businesses in other sectors. Yet generic relationship marketing models such as the one applied in this research do not necessarily reflect these differences (Dibb & Meadows, 2001).

### Customer satisfaction

From consumer’s perspective, the concept of customer satisfaction is useful for giving clearer information about how satisfied or dissatisfied other consumers are with certain products or services. With a more quality information, consumers are expected to be able to make wiser purchasing decisions and can avoid bad experiences of other consumers. Moreover, consumers are also expected to truly understand their position, especially regarding their rights and obligations, as well as the rights and obligations of business actors. In addition to become a reference in evaluating the performance of products and companies, understanding the rights and obligations of consumers and business actors is also useful for giving information about alternative solutions and procedures that can taken if consumers are not satisfied with a specific product/service or company. In this regard, understanding the benefits of customer satisfaction is deemed to be necessary.

Client happiness is universally acknowledged as a critical basis of marketing success, with a happy customer base playing a critical part in a company's competitiveness. In addition, this is contingent on a company's capacity to meet the demands and desires of its target consumers through great product/service performance. In general, satisfaction is defined as a pleasant consequence, a desired end state of consumption, or patronization (Oliver, 1980), whereas customer satisfaction is defined as a collection of beliefs or outcomes related to consumers' experiences with products/services offered (Solomon et al., 2006).

In the services business, Zeithaml et al. (1996) acknowledged that customer satisfaction or discontent is an assessment of a product or service supplied to fulfill the demands or expectations of a client. This is a phenomena that occurs when a client experiences a service and expresses how much they like or detest it (Woodside et al., 1989). As a result, customer satisfaction is the total of a person's perceptions, assessments, and psychological reactions to a product or service's consuming experience (George & Kumar, 2014).

Overall customer satisfaction, on the other hand, is determined by one's assessment of the gap between actual performance/results and customer expectations for the services. The consumer will be satisfied if the performance meets his or her expectations; if the performance exceeds his or her expectations, he or she will be extremely happy and delighted, and vice versa (Kotler et al., 2009).

## Method of study

This study adopted a quantitative approach based on the collection of primary data through the use of a structured survey directed to support a quantification of employees’ perceptions. Selected items and queries proposed by consolidated managerial literature have been used for quantifying each investigated variable. The questionnaire was structured as follows:

- At the beginning, we explained to respondents the purpose of the research. In addition, we also provided them the definitions of Relational Marketing and Customer Satisfaction in several sectors for a better understanding of our survey.
- Second, we asked respondents to assigned a value on a 5-point Likert-type scale (ranging between 1 = absolutely irrelevant and 5 = absolutely important).
- After another focus group with the same management experts which helped us to develop the first items for the first section of the questionnaire, we have identified another items that correspond to the reasons (related to aspects of firms’ intangibles) that have pushed a respondent to participate in one or more activities (if he has participated in past) or that would motivate a respondent to participate (if he has not yet participated). In addition, in this section, we asked respondents to assigned a value on a 5-point Likert-type scale about the importance of these items for them (ranging between 1 = very unimportant and 5 = very important).
- In the final section, we collected socio-demographic data (age, gender, qualification etc.) about the respondents and asked them to provide us other email addresses to contact for our investigation

This study is purposed to examine and analyze the correlation between relationship marketing variables to switching barriers, customer satisfaction, customer trust and customer retention. Regarding to the objectives and study hypotheses, structural equation technique (SEM) is considered as relevant to be used.

The population in this study was BNI customers who are members of BNI Emerald in East Java Province. The sample was obtained based on the top five BNI branches which include the Surabaya Branch, the Graha Pangeran Surabaya Branch, the Tanjung Perak Branch, the Malang Branch and the Gresik Branch. Of the five branches, the total population is 1449 BNI Emerald customers. From calculations using the Slovin formula, a total sample of 141 respondents was obtained. Then, the sample was determined by area with proportional random sampling based on branch.

## Results and discussion

### Goodness of fit model test

Result of the overall model fit (FIT) test shows a large value of 0.729, so that the data variance can be explained by the model of 72.9%, while the remaining 0.271 or 27.1% variance of the data is explained by other variables out of the model. The Adjusted FIT (AFIT) value also shows a decent value of 0.528.203. The two additional measures, namely, the standardized root mean square residual (SRMR), also show a low value closes to 0 and the good fit index (GFI) is very large, namely, 0.996 closes to one. It can be concluded that the model has a good feasibility level (Table 1).

**Table 1:** Goodness of fit model test

Goodness of fit	Value	Conclusions
FIT	0.729	Feasible because it has a large value of 72.9% Model can explain the data
AFIT	0.528	Feasible because it has a large value of 52.8%
GFI	0.996	Feasible because the value is close to 1 (large)
SRMR	0.054	Feasible because the value is close to 0 (small)

*Source* Primary data processed, 2021

### Structural model (inner model)

**Fig. 1:**
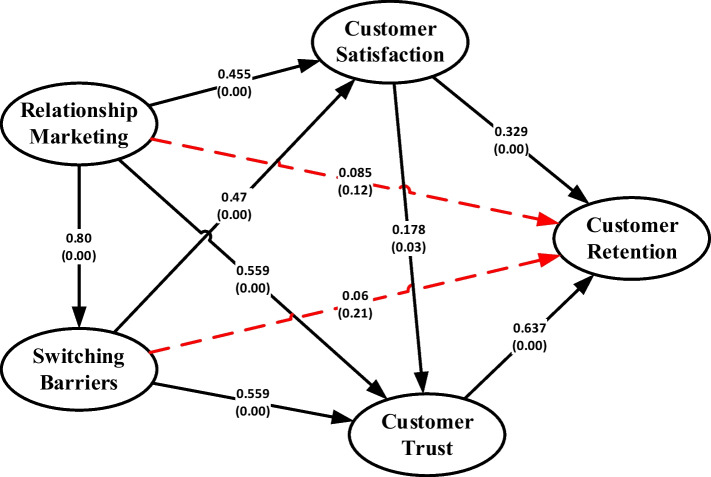
Path analysis results

From the overall test on the correlation inter variables, there are two paths which are not significant, while the other eight paths are significant. Test hypotheses results are presented in the path diagram showed in Fig. 1 (Table 2).

**Table 2:** Path coefficient of the study variables

Correlation intervariables	Coefficient	Std. error	*t* Stat	Sig	Description
Relationship marketing—switching barrier	0.807	0.034	23.73529	0.000	Significant
Relationship marketing-customer satisfaction	0.455	0.081	5.617284	0.00	Significant
Relationship marketing—customer trust	0.559	0.06	9.316667	0.00	Significant
Relationship marketing—customer retention	0.085	0.073	1.164384	0.12	Not significant
Switching barrier—customer satisfaction	0.47	0.077	6.103896	0.00	Significant
Switching barrier—customer trust	0.559	0.06	9.316667	0.00	Significant
Switching barrier—customer retention	0.06	0.075	0.8	0.21	Not significant
Customer satisfaction—customer trust	0.329	0.061	5.393443	0.00	Significant
Customer satisfaction—customer retention	0.178	0.092	1.934783	0.03	Significant
Customer trust—customer retention	0.637	0.129	4.937984	0.00	Significant

*Source* Primary data processed, 2021

### Characteristics of participants

The data on the characteristics of the participants in this study are shown in Table 3. The majority of participants are nonlegality of licensing producers (64.21%), business duration is less than 1 year (42.43%), with the number of employees fewer than 5 people (87.08%) and product variants produced between 1 to 5 kinds of the product (54.24%).

**Table 3:** Characteristics of participants

No.	Information	Number of participants	Percentage (%)
1	Nonlegality of licensing producers	91	62.21
	Legality of licensing producers	50	37.79
2	Business duration is less than 1 year	60	42.43
	Business duration is more than 1 year	81	57.57
3	Employees fewer than 5 people	123	87.08
	Employees more than 5 people	18	12.92
4	Product variants produced between 1 to 5 kinds of the product	76	54.24
	Product variants produced more than 5 kinds of the product	65	45.76

### Hypothesis 1: The effect of relationship marketing on switching barriers

The estimation results of inner model for the direct effect between Relationship Marketing on Switching Barriers show a *t*-statistic value of 23.7352, where it is greater than 1.98, the sig value is 0.000 with an error level of *α* = 5%, meaning that the effect of Relationship Marketing on Switching Barriers is significant. The resulting effect is positive, which means that the better the Relationship Marketing, the more effective the Switching Barrier strategy. This means that the worse the Relationship Marketing, the less effective the Switching barrier strategy will be.

To retain customers, relationship marketing has been developed, because it has a strategic value. In social context, customers need special attention through customer intimacy (by building closeness with customers). The activity of retaining customers through a relationship marketing program gives a signal for priority customers that they have a special position compared to common customers. This treatment acts as a binder for customers to keep their partnership with the company.

### Hypothesis 2: The effect of relationship marketing on customer satisfaction

The estimation results of inner model concerning the direct effect of Relationship Marketing on Customer Satisfaction show a *t*-statistic value of 5.6172, where it is greater than 1.98, the sig value is 0.000 with an error level of *α* = 5%, meaning that the direct effect of Relationship Marketing on Customer Satisfaction is significant. The resulting effect is positive, it means that the better the Relationship Marketing, the higher the customer satisfaction. Conversely, the worse the Relationship Marketing, the lower the customer satisfaction will be.

Satisfaction among various customers is relative; sometimes a customer says that the internet banking services meet his expectations, while others do not. Satisfaction can indeed be subjective. Thus, effective communication which is a part of relationship marketing is absolutely crucial. Customer dissatisfaction with the services can be neutralized by taking a personal approach through several steps such as creating close relationships with intensive communication, giving more attention, facilitating interaction, cooperating with customers, offering rewards and socializing product content.

### Hypothesis 3: The effect of relationship marketing on customer trust

Estimation results of the inner model regarding the direct effect of Relationship Marketing on Customer Trust show a *t*-statistic value of 9.3166, where the value is greater than 1.98, the sig value of 0.000 with an error of *α* = 5%, meaning that the direct effect of Relationship Marketing on customer trust is significant. The resulting effect is positive, which means that the better the Relationship Marketing, the higher the Customer Trust will be. On the contrary, the poorer the Relationship Marketing, the lower the Customer Trust will be.

Basically, banking is a trust business. In building a trust, banks need to create closeness with customers, so that they can communicate and make close and personal interaction with customers. Especially for priority customers who have priority services as promised. Through this good relationship, bank will find it easier to get customer trust thus it will be easier to increase loyalty which ultimately lead to recurring transaction behavior. Zeithaml et al. (1996) suggested that components of Relationship Marketing that are related to customer trust are Reliance on external membership requirements which are defined as trust in members who have special preferences. This of course requires banks to first giving the best service to their existing customers to attract new customers as desired. Banks should be able to satisfy customer expectations, because the satisfied customers will help them promote to other colleagues.

### Hypothesis 4: The effect of relationship marketing on customer retention

Estimation results of the inner model regarding the direct effect of Relationship Marketing on customer retention show a *t*-statistic value of 1.1643, where the value is less than 1.98, the sig value is 0.12 with an error level of *α* = 5%, meaning that the direct effect of Relationship Marketing on Customer Retention is insignificant.

The results show that relationship marketing cannot directly affect customer retention; however, many efforts to build good relationships with customers must have an impact on customer satisfaction and trust as well as become a factor of switching barriers. Banking as a business which lies on trust, repetitive purchasing behavior, and customer loyalty will work after the customer has a satisfactory experience which ultimately form a high sense of trust. Customer retention is the result of a gradual and long-term process. It occurs not because of an instant process, especially in a banking world with its service style. Merely a good relationship is not enough, it must be able to satisfy and build customer trust.

### Hypothesis 5: Effect of switching barriers on customer satisfaction

Estimation results of the inner model concerning the direct effect of switching barriers on customer satisfaction show a *t*-statistic value of 6.1038, where this value is greater than 1.98, the sig value of 0.000 with an error level of *α* = 5%, meaning that the direct effect of Switching Barriers on Customer Satisfaction is significant. The resulting effect is positive, implies that the stronger the Switching barrier, the higher the Customer Satisfaction. On the other hand, the weaker the Switching barrier is, the lower the Customer Satisfaction is.

An effective switching barrier strategy must be implemented by BNI regarding a tight competition among banks. Many efforts such as offering large benefits to customers, personal recognition and personal service need to be specially designed as part of a strategy to bind customers. Many efforts taken by the Bank for retaining its customers and keeping their partnerships with the bank can be positively perceived by customers as an effort to maintain them. Thus, customers will feel satisfied, because they have been positioned as a priority customer whose existence is needed by the Bank. In line with Lovelock & Gummesson (2004) which stated that to get long term benefits, companies must design special steps that are oriented towards increasing attachments through switching barriers which ultimately lead to customer satisfaction.

### Hypothesis 6: Effect of switching barriers on customer trust

Estimation results of the inner model concerning the direct effect of switching barriers towards customer trust show a *t*-statistic value of 9.3166, where the value is greater than 1.98, the sig value is 0.000 with an error level of *α* = 5%, this reflects that the direct effect of switching barrier on customer trust is significant. The resulting effect which is positive gives a sign that the stronger the Switching barrier, the higher the Customer Trust. Conversely, the weaker the Switching barrier, the lower Customer Trust will be.

Bank's efforts to increase the service value to customers can be perceived as a form of high appreciation given, where in the long run, it will impact to customer trust. One of efforts taken to generate customer trust is by consistently fulfilling the promises given to customers, consistent with service and transparency. Regarding to transparency, BNI has to prove its transparency with real performance. In situations of dissatisfaction, customers may make complaints and it usually followed by termination of business relations. Of course this situation must be avoided by banks and various steps must be taken to seek customer sympathy through the concept of switching barriers. One of antecedent factors built by banks is by implementing relationship marketing and switching barriers simultaneously to get customer satisfaction and at the same time create customer trust.

### Hypothesis 7: Effect of switching barriers on customer retention

Estimation results of the inner model for direct effect of switching barriers on customer retention show a *t*-statistic value of 0.8, where it is smaller than 1.977692, the sig value of 0.21 with an error level of *α* = 5%, meaning that the direct effect of switching barrier on Customer retention is insignificant. Thus, the hypothesis stating that there is a correlation between switching barrier on customer retention is rejected.

Findings in this study explain that creating customer retention not merely can be done by creating programs which prevent customers from switching to other service providers. There must be real actions delivered to customers, such as customer satisfaction and customer trust programs. In other words, the switching barrier will only be effective if its program is caught by the customer as an effort to satisfy them and increase their trust. This finding also confirms that switching barrier cannot be the main variable that directly influences it but must go through the intervening variables, namely, customer satisfaction and customer trust. Ranawaera (2003) also noted that switching barrier is more appropriate as a moderating variable that moderates the relationship between customer satisfaction and customer retention, not a direct relationship. Furthermore, Ranawaera suggested that customer satisfaction is not the only antecedent variable to retention. The switching barrier is needed as a reinforcing variable. Basically, Ranawera's statement can be an explanation why the relationship between the switching barrier and customer retention is not directly significant.

### Hypothesis 8: Effect of customer satisfaction on customer trust

Estimation results of the inner model for the direct effect between customer satisfaction on customer trust show a *t*-statistic value of 5.3934, where it is greater than 1.98, the sig value is 0.000 with an error level of *α* = 5%, meaning that the direct effect of customer satisfaction on customer trust is significant. The resulting effect is positive, which means that the stronger customer satisfaction, the higher the customer trust. On the other hand, the lesser the customer satisfaction, the lower the customer trust will be.

The satisfied customers on company's services will show behavior that benefits the company, including increasing customer trust and increasing customer loyalty, followed by repeated purchasing behavior (customer retention). Company’s success in satisfying its customer will also reduce customer sensitivity to the consequences of costs as well as time and energy generated during transaction activities with the company. The success can also gives impact to the reputation of the company. Several indicators of customer satisfaction that can be considered by BNI are related to efforts to increase customer trust, such as improving customer service, creating a pleasant service impression and easy transaction processes, and providing supportive facilities.

### Hypothesis 9: Effect of customer satisfaction on customer retention

Estimation results of the inner model regarding the direct effect of customer satisfaction towards customer retention show a *t*-statistic value of 1.9347, where it is greater than 1.98 but the sig value of 0.03 is below the error level *α* = 5%, meaning that the direct effect of customer satisfaction on customer retention is significant. The resulting effect is positive, which means that the stronger the customer satisfaction, the higher the customer retention. Conversely, the weaker the customer satisfaction, the lower the customer retention.

According to several previous studies, the satisfied customers will behave in a way that benefits the company. Some of the behaviors are including commitment to become a customer, willingness to cooperate profitably, increase transaction volume and strengthen emotional ties. Customer relations have a moderating function towards the relationship between satisfaction and customer retention. BNI Emerald customers are those who are prioritized to be served with flexibility and special treatment. BNI must be consistent with its customer satisfaction program by always improving its service quality standards. The service excellence jargon that is always used by banking services in acquiring customers must be proven and the benefits for customers can be felt.

### Hypothesis 10: Effect of customer trust on customer retention

Estimation results of the inner model regarding the direct effect of customer trust on customer retention show a *t*-statistic value of 4.9379, where it is greater than 1.98, the sig value is 0.000 with an error level of *α* = 5%, meaning that the direct effect of customer trust on customer retention is significant. The resulting effect is positive, which means that the stronger the customer trust, the higher the customer retention. Likewise, the poorer the customer trust, the lower the customer retention will be.

Customer retention behavior is the output of customer trust as a result of good relationships with customers and the Bank success for consistently providing optimal customer value. Customer trust is a dynamic thing, so it must be maintained through superior, cooperative and more customer-oriented service fulfillment. Customers with high trust on the bank will become strongly committed customers, have willingness to mutually cooperate with the bank, increase the transactions volume and forge stronger emotional bonds.

### Hypothesis 11: The indirect effect of Relationship Marketing on Customer Retention

From the path analysis of the research model which connecting the effect of relationship marketing to customer retention through switching barriers, customer satisfaction and customer trust, it is known that the path with the greatest coefficient is the relationship marketing influence path on customer retention through customer trust variables with a total coefficient value of 0.34. Other path with the second greatest value is the effect of relationship marketing on customer retention through customer satisfaction with a total coefficient value of 0.14. Thus, it can be concluded that trust is more dominant than satisfaction as the mediating variable between relationship marketing variable and customer retention variable. This finding is also consistent with the partial analysis which showing that the effect of the trust variable on customer retention has the greatest coefficient value (0.637, sig. 0.00) followed by satisfaction with the second largest coefficient value (0.329, sig. 0.00).

It has been previously mentioned that customers interact with the bank in terms of making investment. A customer who has invested an asset expects that the asset will be managed properly to get a return or other benefits. This is where the exchange process occurs between the two parties, each of which has different motivations. The bank will get more benefits when customer funds remain in the bank for a long period. However, the consequence is the bank must keep the customer trust towards the bank itself.

## Discussion

The shift in marketing viewpoint from an internal (inward-looking) to an external (outward-looking) orientation has prompted this research to investigate the notion of relational marketing in greater depth. Because of its strategic importance, the notion of relationship marketing is now being researched extensively in the fields of service marketing and industry. As a result, this research study on relational marketing must be completed as soon as possible to corroborate the findings of prior relevant marketing research. According to this study, the ideal marketing strategy is to keep existing consumers rather than gain new ones. Finally, this concept inspired scholars to investigate relationship marketing. The fundamental concept of relational marketing is a paradigm shift away from transactional marketing and toward long-term consumer connections. According to Mulyana (2019), the company’s ability to understand customer needs and respond to competing strategies for market segments that are committed to products with religious values and supported by religious-based marketing strategies will potentially increase marketing performance.

Trust defines the willingness to have confidence in a partner who one can rely on with certainty. The transmission of pertinent and timely information, whether official or unofficial, between buyers and sellers is referred to as the communication component. One can view a situation from the perspective of other individuals thanks to the empathy component. The fourth factor is shared values, which explains how strongly spouses agree on rules, conduct, and appropriateness (Morgan & Hunt, 1994). Reciprocity highlights a procedure that enables the customer to connect with and share data with the company, enabling it to address the needs of the customers (Jayachandran et al., 2005). The final aspect is bonding, which describes emotional development between two entities that act cooperatively for a common goal (Sin et al., 2005). In this sense, relationship marketing orientation may function as an influencer. The inclination of the connection in corporate social responsibility suppliers and customer value co-creation may be increased by the relevant actions of the firm. Relationship marketing strategies can encourage suppliers to forge stronger ties with the company and its commitment to CSR, as well as customers to take an active role in the process (McColl-Kennedy et al., 2012; Tse et al., 2004). The relationship marketing orientation is an additional investment made by businesses to deepen their connections with customers to co-create value, which is also a kind of reciprocity and the result of relationship strategy (Tse et al., 2004). Every aspect of relationship marketing orientation promotes customers' impressions of the company's values in terms of corporate social responsibility, which ultimately leverages the customers' reciprocal contribution in terms of value co-creation activity.

Several prior research, including Dwayne Ball (2004), Ranaweera (2003), Sahadev (2011), Vatanasombut Banphot et al. (2008), and Liu et al. (2011), have demonstrated that customer satisfaction is a significant factor in establishing customer loyalty and retention. The analysis is expanded in this research by include relational marketing as an antecedent variable that influences customer happiness and retention. Palmatier et al. (2006) concluded in their meta-analysis that the essence of a relationship between a customer and a seller should be captured in a sparse net, beyond trust and commitment alone. In their meta-analysis, Palmatier et al. (2006) came to the conclusion that, beyond trust and commitment alone, the core of a connection between a consumer and a supplier should be recorded in a sparse net. This idea is illustrated in the current study, where trust made the smallest contribution to the indicator of relationship quality (although the difference between the indicators was marginal). Because there is less perceived risk in low participation marketplaces, customers may place more emphasis on the now (i.e., satisfaction) than the future fulfillment (Garbarino & Johnson, 1999).

While these findings pertain to consumer markets, it is possible to envisage a similar problem in business-to-business markets, where loyalty programs become more prevalent. The recent analysis indicates that despite the numerous promises made by loyalty programs, customers do not perceive any added value (Kwiatek et al., 2020). The findings of this study indicate that if customers are actively engaged in a loyalty program, concrete results can be attained (i.e., they redeem points). In other words, the reciprocity in a relationship is brought about by the fulfillment of a promise made in a loyalty program (getting a reward). The study's findings also imply that compensating clients to encourage referrals would not be a workable option in a transactional market. A favorable assessment of a provider leads to a willingness to recommend them (a preference for a focal supplier). According to study by Mubushar (2020), customer value co-creation behavior is positively impacted by both supplier-related and local community-related CSR initiatives. The relationship between CSR activities and customer value co-creation behavior is mediated by relationship marketing orientation. RMO is observed to have a more dominant role in CSR supplier and customer value co-creation behavior.

Many marketing scholars studied the strategic importance of service quality as the major component that leads to the production of business profits several decades ago. This research takes a different approach, arguing that the capacity to build solid customer connections is what businesses need to safeguard their long-term earnings. As a result, relationship marketing will be investigated in conjunction with other essential factors, such as switching barrier variables, customer happiness, customer trust, and customer retention in this research. Marketing and financial performance have two antecedents: brand orientation and inter-firm market. When the brand orientation is positive, the inter-firm market has a substantial impact on marketing and financial performance. Previous studies have looked at the moderating effect of brand orientation on interfirm market orientation, which is crucial, especially for companies looking to cooperate with other companies to boost their brand reputation. To understand why maintaining a strong brand presence is crucial in the global marketplace and the key modifiers of the driving force of the inter-firm market in connection to company performance, more research is recommended (Tajeddini & Ratten, 2017).

The inclusion of various relationship marketing factors, such as switching obstacles, customer happiness, customer trust, and customer retention, is another methodological innovation in this research. Many studies in the service industry, particularly banking, use regular customers as units of research. The conduct of BNI Emerald consumers is the subject of this investigation. Because it is tough to engage clients who are classed as premium class, this customer segment is still understudied. The discovery of a study model that has been proven by SEM analysis test that the relationship marketing variables and switching barriers are not 237 pure exogenous variables is the last note that can be inferred as an uniqueness of this research. By involving the factors of customer trust and customer pleasure, the existence of these two variables will demonstrate their importance.

## Conclusions

This study extends previous research by including relational marketing as an antecedent variable that affects customer satisfaction and retention. As a result, relational marketing will be investigated along with other important factors, such as switching barrier variables, customer happiness, customer trust, and customer retention in this research. The inclusion of various relational marketing factors, such as switching barriers, customer happiness, customer trust, and customer retention, is another methodological innovation in this study. As a result, relational marketing is positioned as the primary exogenous variable to be investigated in conjunction with other relevant factors, such as switching barrier variables, customer happiness, customer trust, and customer retention. Customer satisfaction has a considerable positive impact on customer trust, indicating that the better the customer satisfaction, the higher the customer trust. Customer satisfaction has a positive and considerable impact on customer retention, indicating that the better the customer satisfaction, the higher the customer retention.

## Data Availability

On request, the corresponding author can provide the data used in the paper. The questionnaire was designed using selected items and inquiries offered by aggregated management literature for measuring each researched variable. We asked respondents to rate their importance on a 5-point Likert scale (with 1 being completely unimportant and 5 being completely vital).
